# Examining transmission of gut bacteria to preserved carcass via anal secretions in *Nicrophorus defodiens*

**DOI:** 10.1371/journal.pone.0225711

**Published:** 2019-12-02

**Authors:** Christopher James Miller, Scott Thomas Bates, Lindsay M. Gielda, J. Curtis Creighton

**Affiliations:** 1 Department of Biological Sciences, Purdue University Northwest, Hammond, IN, United States of America; 2 Department of Biological Sciences, Purdue University Northwest, Westville, IN, United States of America; Universiteit Leiden, NETHERLANDS

## Abstract

Direct transmission of bacteria to subsequent generations highlights the beneficial nature of host-bacteria relationships. In insects, this process is often mediated by the production of microbe-containing secretions. The objective of this study was to determine if the burying beetle, *Nicrophorus defodiens*, utilizes anal secretions to transmit adult digestive tract bacteria onto a small vertebrate carcass; thus creating the potential to aid in carcass preservation or pass digestive tract bacteria to their larval offspring. Using high-throughput Illumina sequencing of the 16S rRNA gene, we characterized bacterial communities of adult beetle digestive tracts, their anal secretions, and prepared mouse carcasses. We also examined unprepared carcass bacterial communities as a means to interpret community shifts that take place during carcass preservation. We found a vast reduction in diversity on prepared carcasses after anal secretion application. Overall, there was little similarity in bacterial communities among adult digestive tracts, anal secretions, and prepared carcasses, suggesting bacterial communities found in adult digestive tracts do not successfully colonize and achieve dominance on prepared carcasses by way of beetle anal secretions. We concluded that *N*. *defodiens* does not transmit their digestive tract bacterial communities to prepared carcasses in a wholesale manner, but may transmit key microbes, including core microbiome members, to preserved carcasses that may ultimately act to sustain larvae and serve as inocula for larval digestive tracts.

## Introduction

Microbes provide beneficial and sometimes essential functions for their hosts. For example, they may aid in development [1–3], assist in digestive processes and nutrient acquisition [4–6], act as an innate defensive barrier against pathogens [7–9], and prime the host immune system [10–12]. Host-microbial relationships can also drive the creation and/or preservation of food sources. For example, *Paramecium bursaria* maintains endosymbiotic *Chlorella*-like microalgae that provide the protist with photosynthates, thus diminishing its need for external sources of nourishment [13]. Leafcutter ants (*Acromyrex octospinosus*) cultivate fungal gardens in their colonies as a food source, [14] inoculating antimicrobial-producing bacteria onto their fungal cultures to preserve and protect this resource from other microbial competitors [15]. Insects can also use antimicrobial compounds to preserve food sources for developing larvae, which is seen as an effective parenting strategy [16]. European beewolves (*Philanthus traingulum*) provision paralyzed honeybees to developing larvae as food and deposit antimicrobial compounds on to the bees to inhibit fungal growth and subsequent carcass decomposition [17]. In this study, we evaluate the role parental anal secretions play in transferring bacteria to their offspring food source in the burying beetle *Nicrophorus defodiens*.

Burying beetles use a small vertebrate carcass as the food source for their developing larvae. When burying beetles discover a carcass, it is buried underground, stripped of fur or feathers, rolled into a ball, and covered with oral and anal secretions [18–22]. The oral and anal secretions contain antimicrobial compounds that can preserve the carcass by eliminating decomposing microbes [22,23]. Recent research has examined the role of microbes found in the secretions of *Nicrophorus* species, which are also transferred onto the carcass [16,24–26]. Anal secretions produced by *N*. *vespilloides* have been shown to control the carcass microbiome by promoting the growth of *Yarrowia*, an oleaginous fungal yeast, and inhibiting growth of other microbial species [24,26]. Controlling the carcass microbiome in this way provides larvae prolonged and easy access to carcass nutrients, increasing survivorship [16,25]. Although the bacterial taxa of prepared carcasses, anal secretions and adult digestive tracts are all thought to be similar [18,25], the role *Nicrophorus* anal secretions play in directly shaping bacterial communities of the carcass, which can ultimately serve as inocula for the larval gut community, remains unclear.

The precedent of fungal transmission onto the carcass via anal secretions by *N*. *vespilloides* suggest burying beetles may also transmit bacteria to the prepared carcass in order to influence the microbial community there. This process, however, has never been investigated in other *Nicrophorus* species. In this study, we used high-throughput sequencing to characterize bacterial communities found in adult *N*. *defodiens* digestive tracts, in their anal secretions, and on prepared as well as unprepared carcasses in order to evaluate: 1) the role anal secretions might play in shaping the bacterial communities of prepared carcasses; and 2) the extent that anal secretion or carcass bacterial communities retain the adult burying beetle gut bacteria to act as potential inocula for the larval offspring gut flora.

## Materials and methods

### Population maintenance and sample collection

Baited pitfall traps were used to collect *N*. *defodiens* during June and August of 2017 in Big Falls, Wisconsin. Captured beetles were used to establish the laboratory population for our study, in which only F1 and F2 beetles were used. Laboratory populations were kept individually in small plastic (7 x 7 x 5 cm) containers with a small moist paper towel. All beetles were kept in an environmental chamber set to 20°C with a 14:10 hour light:dark cycle [27] and fed chicken liver twice weekly [28]. Upon reaching sexual maturity, roughly 15–25 days after eclosion, mating pairs were placed in large, sterilized plastic (18 x 15 x 10 cm) containers (“brood chambers") with a freshly thawed 15–20 g mouse carcass on top of 4–5 inches of freshly autoclaved commercial topsoil.

Samples from unprepared carcasses were taken by swabbing all external surfaces with a sterile cotton swab dipped in sterile PBS immediately after thawing. Forty-eight hours after beginning the reproductive bout, prepared carcasses were swabbed on all external surfaces and within feeding holes using a sterile cotton swab dipped in sterile PBS. Prepared mouse carcasses were defined as those that were fully or partially buried, rolled into a ball, having all their hair removed, and having an observational presence of anal secretions [18]. Cotton swab tips were placed in 1.5 mL Eppendorf tubes, submerged in pure sterile glycerol, and stored at -80°C until needed for DNA extraction [29].

At the time prepared carcasses were sampled, females were removed from brood chambers and the anal secretions were collected. Because beetles drag their posteriors across the carcass and soil when they apply anal secretions to a carcass, these areas were thoroughly surface sterilized with 70% isopropanol wipes to eliminate external contamination. Beetles were then air dried, and anal secretions were collected by gently pressing a sterile capillary tube directly onto the posterior area. Anal secretions were transferred to 0.5 mL Eppendorf tubes, diluted 1:5 with sterile PBS, and stored at -80°C until needed for DNA extraction [30]. Once anal secretions were collected from females, all insects were prepared for dissection (see details of this process below).

### Dissection

All beetles were dissected (see below) immediately after removal from brood chambers in order to minimize the effect of sudden environmental changes on digestive tract bacterial communities [16]. Beetles were surface sterilized by rinsing with 70% ethanol twice and then again with sterilized diH_2_0 to eliminate any contamination from the soil, carcass, or exoskeleton. Specimens were euthanized by decapitation using sterile fine point scissors [26], and body cavities were dissected by creating an incision at anterior end and cutting down the side of the beetle towards the posterior using sterile fine point scissors. Sterile insect pins were used to open body cavities and the complete digestive tract was removed using sterile fine point forceps. Digestive tracts were placed in 0.5 mL Eppendorf tubes, submerged in pure sterile glycerol, and stored at -80°C until needed for DNA extraction [29].

### DNA extraction and high throughput sequencing

DNA was extracted from the stored cotton swab tips using a DNeasy Power Soil DNA Extraction Kit (Qiagen, Venlo, Netherlands). Cotton swab tips were removed with sterile scissors and then added directly to the kit beaded tube, after which the standard kit protocol from the manufacturer was followed. DNA was extracted from the dissected adult digestive tracts using the same DNeasy Power Soil DNA Extraction Kit. The manufacturer’s protocol was followed with the addition of a preliminary 10-minute heating step at 75°C prior to beaded tube vortexing, which assisted in degradation of the intestinal tissue. DNA from anal secretion samples was extracted using a DNeasy Blood and Tissue DNA Extraction Kit (Qiagen, Venlo, Netherlands) following the standard kit protocol. Pure DNA extracts were sent to the University of Colorado, Boulder for high throughput Illumina sequencing on the MiSeq platform following previously described methods [30].

### Bioinformatic processing and statistical analysis

Barcoded sequences were imported into Quantitative Insights into Microbial Ecology v.2.4 (QIIME2) [31]. Single-end reads were demultiplexed, chimeric sequences were removed, and samples were subsequently de-noised using the Deblur pipeline [32,33]. Samples were rarefied to a depth of 5,000 sequence reads per sample and operational taxonomic units (OTUs) were assigned using the Greengenes 13.8 reference database with an 88% sequence similarity [33,34]. Taxa bar plots were generated using the q2-feature-classifier plugin [34].

All subsequent statistical analyses and calculation of diversity metrics were carried out using QIIME2 [32] or R software v.3.4.3 (https://www.r-project.org/) and the Vegan package. Bray-Curtis dissimilarity distance matrices were utilized for ordination and Analysis of Similarities (ANOSIM) performed with 999 free permutations. ANOSIM produces an R statistic ranging from -1–1 where values closer to 0 represent a more similar relationship between bacterial communities and values further from 0 represent a more dissimilar relationship [35]. Permutational multivariate analysis of variance (PERMANOVA) also used 999 free permutations and was performed in R v.3.4.3. PERMANOVA produces a pseudo-F value that when closer to 0, represents more similar bacterial communities and when further from 0, represents more dissimilar communities. Post-hoc PERMANOVA pairwise analyses were conducted with Bonferroni corrections in R v.3.4.3.

### Ethics statement

This research was conducted in compliance with internationally accepted standards for the ethical treatment of animals. Since the subject matter of this research involved insects and commercially purchased frozen mice, IACUC approval was not required. All beetles used in this study were collected on the private property of Jane and Stefan Shoup (Big Falls, Wisconsin, US), who provided explicit permission to perform pitfall trapping on their privately owned property.

## Results

### High throughput sequencing

Quality filtering and chimera removal resulted in 677,802 high quality sequence reads, with each of the 50 samples having 13,557 (± 4,348) bacterial sequences on average. Two extraction-blank samples contained less than 150 sequences each, suggesting minimal contamination, and these sequence types were removed from all subsequent analyses. All samples were then rarefied to a sampling depth of 5,000 sequences. Rarefaction analysis (S1 Fig) showed the collector’s curve saturated at a sampling depth of ca. 4,500 sequences, suggesting that rarefaction to a depth of 5,000 sequences captured the full breadth of bacterial diversity our samples. Clustering recovered an assembly of 605 distinct OTUs, however, OTUs not represented by 50 sequences or more were removed from subsequent analyses due to their potential to be sequencing artifacts [36].

### Taxonomic assignments

**Fig 1:**
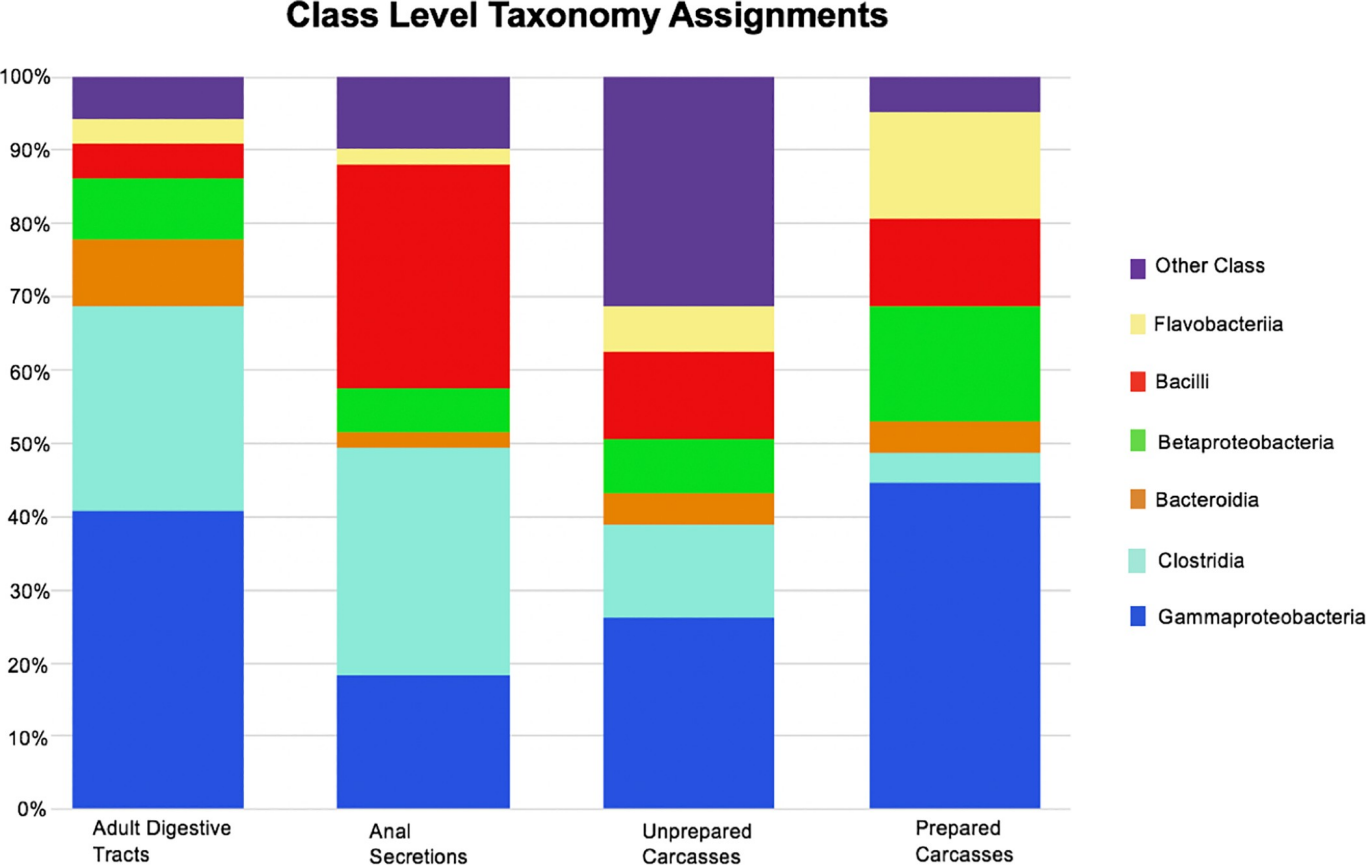
Relative abundances of bacterial classes within different sample types. Bar lengths show the average relative percentage of all sequences for bacterial classes found within each sample type. All classes not specifically identified were assigned to the ‘Other’ category that also includes unidentified bacterial classes.

Each sampling type was dominated by bacterial genera in two classes of bacteria (Fig 1 and S2 Fig). The dominant classes for adult digestive tracts were *Gammaproteobacteria* (41% of all sequences) and *Clostridia* (28%). The *Gammaproteobacteria* (45%) and *Betaproteobacteria* (16%) dominated prepared carcasses, while the most dominant classes for unprepared carcasses were *Gammaproteobacteria* (26%) and *Clostridia* (13%). Dominance in the anal secretions differed slightly from the other sample types, with the dominant classes in this sample type being *Bacilli* (30%) and *Clostridia* (28%).

Genus-level relative abundances of the prevalent (abundance > 10%) bacterial groups differed among all sample types (Table 1, S2 Fig). Adult digestive tracts had three prevalent bacterial groups: *Wohlfahrtiimonas* (13.3%), unidentified Gammaproteobacteria (10.4%), and *Clostridium* (10.2%, Table 1). Anal secretions also had three bacterial groups with relative abundances greater than 10%: *Planococcus* (13.3%), *Tissierella* (10.4%), and *Lactobacillus* (10.0%). Unprepared carcasses, which exhibited the greatest overall diversity, had only one dominant bacterial group, *Acinetobacter* (10.5%), with a relative abundance greater than 10%, while the prepared carcasses had two, *Acinetobacter* (24.8%) and *Vitreoscilla* (14.3%).

**Table 1:** Percentage of prevalent bacterial taxa found across sample types. Taxonomic assignments of the most common bacterial genera found in each sample type, with their class-level designation given in parentheses. A relative abundance threshold of > 10% of all sequences was used here for taxa considered to be ‘prevalent’; however, values lower than 10% also appear for comparative purposes. Bolded percentages represent the three most abundant bacterial groups within a given sample type, and the bottom row shows the total percentage of all sequences within a given sample type. Acronyms are as follows: DT–Adult digestive tract, AS–Anal secretions, UC–Unprepared carcasses, PC–Prepared carcasses.

Taxon	Sample Type			
	DT	AS	UC	PC
*Wohlfahrtiimonas* (Gammaproteobacteria)	**13.3**	5.7	**2.7**	5.9
Unidentified Gammaproteobacteria	**10.4**	1.0	0.1	<0.1
*Clostridium* (Clostridia)	**10.2**	2.1	<0.01	<0.01
*Tissierella* (Clostridia)	7.4	**10.4**	0.4	0.3
*Acinetobacter* (Gammaproteobacteria)	6.8	9.0	**10.5**	**24.8**
*Vitreoscilla* (Betaproteobacteria)	5.6	4.3	**3.9**	**14.3**
*Dysgonomonas* (Bacteroidetes)	5.1	0.7	0.6	2.1
*Planococcus* (Bacilli)	0.8	**13.3**	1.8	**9.2**
*Lactobacillus* (Bacilli)	2.4	**10.0**	0.1	0.6
*Ruminococcus* (Clostridia)	2.4	6.2	0.3	0.1
*Peptoniphilus* (Clostridia)	1.4	5.1	0.6	2.7
*Myroides* (Flavobacteria)	1.3	1.0	2.1	8.9
**Total Representation**	**69.5**	**68.8**	**23.2**	**69.1**

### Alpha and beta diversity

**Fig 2:**
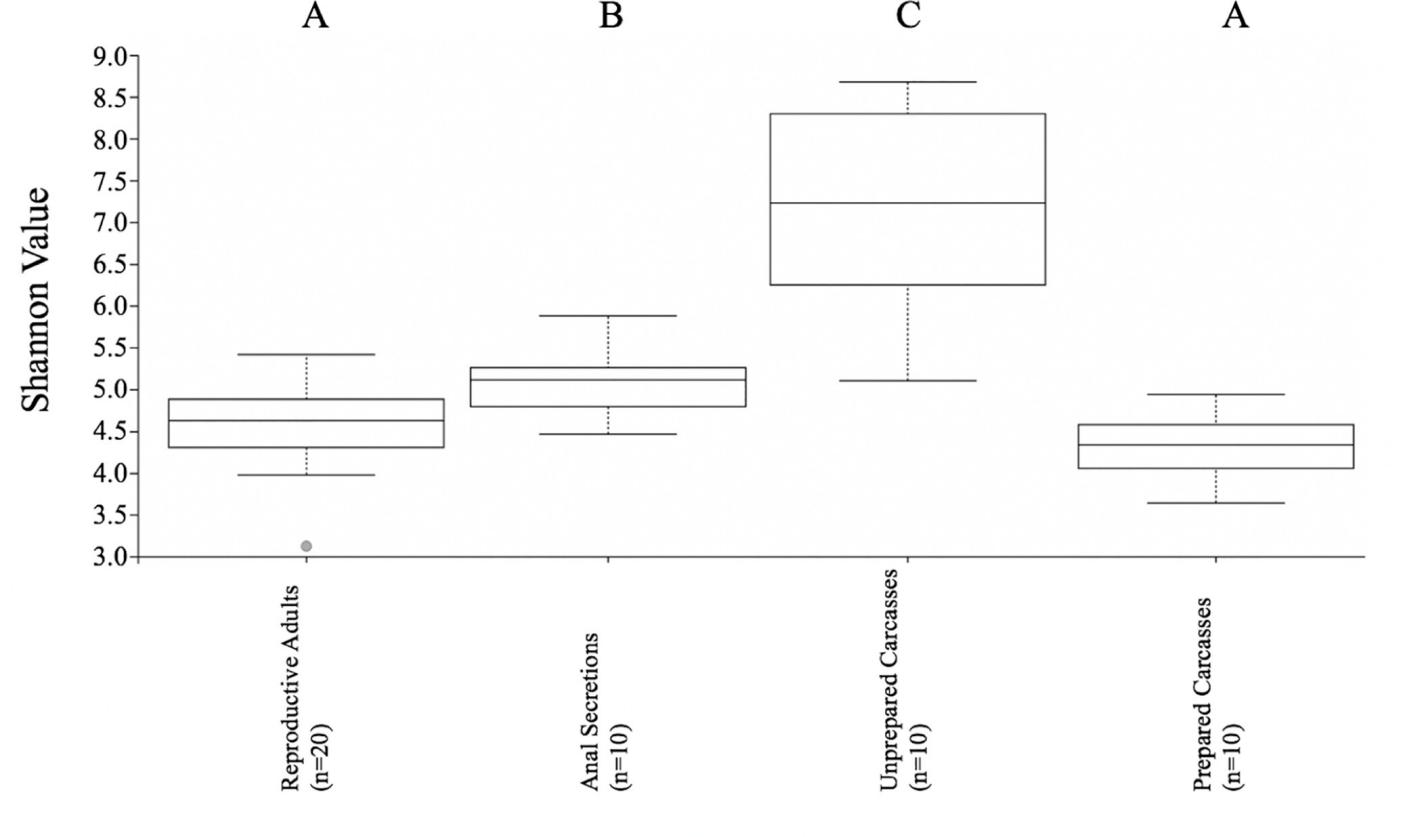
Sample alpha diversity. Box plots depict Shannon-Weiner values compared with pairwise Kruskal-Wallis analysis. Letters designate significantly different groups (*p* < 0.05).

**Fig 3:**
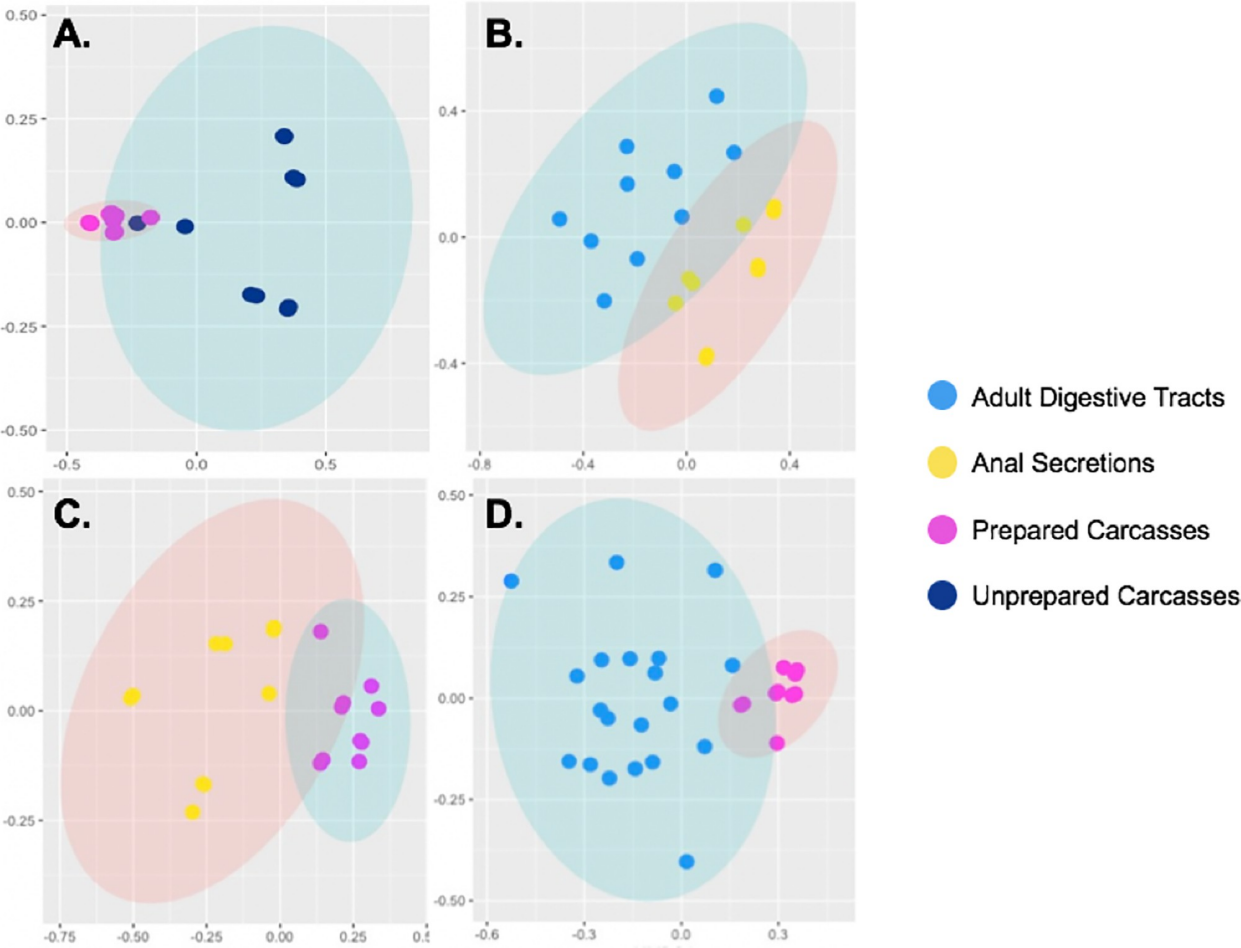
Detrended correspondence analysis (DCA) of sample bacterial communities. Ordination plots depicting significant different clustering patterns (ANOSIM *p* < 0.05) between: A) unprepared vs. prepared carcasses, B) adult digestive tracts vs. the anal secretions, C) anal secretions vs. prepared carcasses, and D) adult digestive tracts vs. prepared carcasses.

Alpha diversity measures for all sample types are provided in Table 2 and S1 Table. Unprepared carcasses had the highest average richness values, with 386 OTUs, while after preparation, the average carcass diversity dropped considerably to just 71 OTUs. Adult digestive tracts and the anal secretions had a similar average richness values, with 86 and 80 OTUs recovered from these sample types, respectively. Shannon-Weiner index values (Fig 2, Table 2), Chao1, Evenness, and Faith’s Phylogenetic Diversity showed similar trends (S1 Table). Distinct clustered patterns were seen in the ordination, with bacterial communities separating significantly among the various sample types (Fig 3). ANOSIM analysis indicated that differences in adult digestive tract bacterial communities between the sexes were not significant (R statistic = -0.062; *p* = 0.77), suggesting there is a common adult gut flora; however, bacterial communities among the other sample types were significantly different overall (R statistic = 0.610; *p* = 0.001). When individually compared (Fig 3), bacterial communities were significantly different between: unprepared vs. prepared carcasses (R statistic = 0.458; *p* = 0.031), adult digestive tracts vs. their anal secretions (R statistic = 0.553; *p* = 0.001), anal secretions vs. prepared carcasses (R statistic = 0.779; *p* = 0.001), and adult digestive tracts vs. prepared carcasses (R statistic = 0.588, *p* = 0.001). Post hoc pairwise PERMANOVA with Bonferroni corrections confirmed that bacterial communities between all sample types were significantly different (S2 Table).

**Table 2:** Average alpha diversity within sample types. Standard richness and Shannon-Wiener Index values are provided with standard error. Acronyms are as follows: DT–Adult digestive tracts, AS–Anal secretions, UC–Unprepared carcasses, PC–Prepared carcasses.

	Sample Type			
	DT	AS	UC	PC
Standard Richness	86 ± 8	80 ± 11	386 ± 23	71 ± 7
Shannon-Wiener Index	4.65 ± 0.09	5.10 ± 0.13	7.38 ± 0.39	4.47 ± 0.11

## Discussion

In this study, we used culture-independent methods to evaluate the potential for anal secretions to shape bacterial communities of prepared carcasses, and ultimately act as inocula for the larval gut flora. Specifically, we utilized high-throughput sequencing to examine the degree to which the adult gut bacterial communities remain in their anal secretions and on prepared carcasses. To the best of our knowledge, this is the first study for *N*. *defodiens* that specifically addresses the role of anal secretions in influencing the structure of the carcass bacterial community and in transmitting bacteria from adult beetle digestive tracts to carcasses by comparing bacterial communities across the adult digestive tracts, their anal secretions, as well as unprepared and prepared carcasses.

We found there was a significant reduction in diversity between the unprepared and prepared carcasses, which is consistent with previous reports [16,18,24] and further implies microbial diversity is controlled in the process of carcass preparation. Despite an overall decrease in the diversity on prepared carcasses, we found that some bacterial taxa (*Acinetobacter*, *Myroides*, *Peptonophilus*, *Planococcus*, *Vitreoscilla*, and *Wohlfahrtiimonas*) increased in abundance on prepared carcasses relative to unprepared carcasses (Table 1). None of these bacterial groups were dominant across all sample types and their abundances varied considerably across the different sample types. The general lack of sustained dominance of these bacterial taxa across sample types suggests that anal secretions do not universally control the carcass bacterial community to favor prevalent gut bacteria as is seen, for example, with some fungi [15]. We were unable to determine if the increase in relative abundance of certain bacterial taxa on prepared carcasses was the result of deposition by anal secretions or reduced bacterial competition. Further, it remains unknown if alterations in bacterial relative abundances are the result of carcasses being exposed to conditions of the environmental chamber for 48 hours or the effects of beetle activity, however the latter seems more likely. Additionally, the soil used in our study was sterilized prior to setting up beetle mating pairs on carcasses. In natural environmental settings, the possibility exists that soil microbes may play a role in further shaping the bacterial community of a prepared carcass. Further studies are needed to determine the influence soil microbes play in shaping these bacterial communities.

Increase in abundance of specific bacterial taxa on prepared carcasses suggests they may have importance as inocula for larval digestive tracts and carcass preparation did selectively favors some adult digestive tract bacteria, such as *Acinetobacter* and *Vitreoscilla*, over others. These taxa were highly enriched on prepared carcasses and were also among the few taxa that sustained a moderate level of abundance across the sample types. This suggests that while there is no overall control of prepared carcass bacterial communities, some bacteria may be directly transmitted from adult digestive tracts to their anal secretions and ultimately onto prepared carcasses, where they have the potential to serve inocula to shape the larval gut flora. We also noted that some adult digestive tract bacteria were depleted in their anal secretions and on prepared carcasses. The antibiotic qualities of anal secretions may play a role in eliminating some, potentially nonessential, digestive tract bacteria from carcasses. For example, *Clostridium* (*C*. *colinum*), was dominant in the adult digestive tract (>10%), but was highly reduced in their anal secretions (~2%) and hardly detectable on prepared carcasses (<0.01%). As *C*. *colinum* is a known contaminant of poultry, we suspect that this species was highly abundant in adult digestive tracts as a byproduct of the chicken liver fed to the beetles. Overall, these observed patterns are generally consistent with the concepts of microbial ‘seeding’ and 'weeding' of the carcass community, as has been show for *N*. *vespilloides* [18], with anal secretions playing an integral role in these processes. Other authors have also postulated that taxa of lower abundance within microbial communities can hold the potential to later become more dominant members through processes such as microbial ‘blooms’ [37], and may be important for controlling developing communities of offspring [38,39]. Further exploration is required to determine if favorable selection of particular taxa on to prepared carcasses results in the ultimate successful inoculation into larval digestive tracts.

Bacterial communities within the adult digestive tracts were significantly different from those found in their anal secretions, however, there were some class-level structural similarities, particularly the high relative abundances of Clostridia and Gammaproteobacteria. Interestingly, the three most prevalent bacteria taxa in the adult digestive tract, *Clostridium*, unidentified Gammaproteobacteria, and *Wohlfahrtiimonas*, belonged to these classes and were each represented within the anal secretion bacterial communities, although at much lower relative abundances. All but one of these taxa (*Wohlfahrtiimonas*) were nearly undetectable on prepared carcasses, indicating that microbial seeding of carcasses is not a direct means of transmission for some of the dominant bacteria of the adult digestive tract. Similarly, a number of less prevalent taxa from the adult digestive tract were found in their anal secretions, with some of these (*Tissierella*, *Planococcus*, and *Lactobacillus*) being enriched for in the anal secretion bacterial communities. These bacteria, however, were typically of fairly low abundance in the adult digestive tract or on prepared carcasses, again pointing to the fact that microbial seeding may not be a successful strategy for generally transmitting all digestive tract microbiota and suggesting that at least some of the patterns seen here may be the result of random processes. Further, while the prevalence of *Planococcus* in the prepared carcass bacterial community may imply its importance as a ‘seeding’ population; the very low (< 1%) relative abundance of this taxon in the adult digestive tract speaks against this. We cannot, however, rule out the possibility that such taxa may be of importance in the larval gut community and later diminish in abundance as the insect matures.

Despite the fact that the overall bacterial community structure transitioned significantly from adult digestive tracts, to their anal secretions, and onto the preserved carcasses, it was changes in the relative abundances of particular bacterial taxa that primarily drove these patterns, and we did recover a core group of bacteria present in all sample types. Bacterial taxa detected in our study, such as *Acinetobacter*, *Dysgonomonas*, *Myroides*, *Vitreoscilla*, and *Wohlfahrtiimonas*, were among this group that previous studies have identified as being associated with *Nicrophorus* spp. [16,18,24–26,40]; thus, suggesting these bacteria are core members of a general *Nicrophorus* microbiome consortia. Many of these potential core microbes have also been isolated from digestive tracts of other insects and have previously been shown to carry out several metabolic functions that hold relevance for the concept of carcass preservation for feeding larval offspring. For example, *Wohlfahrtiimonas* spp. have been isolated from digestive tracts of *N*. *vespilloides*, *Diptera*, and the Rocky Mountain Wood Tick [18,25,41,42] and they are capable of breaking down a variety of amino acids, fermenting a myriad of sugars, and reducing nitrate [42,43]. *Dysgonomonas* spp. have been isolated from carcasses prepared by burying beetles [16] as well as in the digestive tracts of other insects [44–46], and they have the ability to degrade a variety of fatty acids. *Acinetobacter* spp. are capable of producing biofilms and exogenous enzymes that degrade vertebrate tissue [47], while *Myroides* spp. have previously been detected in the digestive tract of burying beetles and on prepared carcasses [18,23,40] and are also known to produce a variety of antibacterial substances [48,49]. *Vitreoscilla* spp., which have also been found previously on carcasses prepared by burying beetles [16,24], have the capacity to metabolize toxic chemicals [50] and have genes likely involved in gastrointestinal tract colonization [51]. Taken together, these data suggest roles for these potential core microbiota in eliminating microbial competitors for carcass preservation, facilitating nutrient acquisition for developing larvae through the breakdown of animal tissue, detoxifying the carcass environment, and even in assisting with re-introduction of core bacteria into the larval digestive tract.

The major shifts in microbial community structure observed in our study are perhaps not surprising given the highly variable conditions that bacteria in these populations experience as they transition from the adult gut, to the anal secretions, and on to the prepared carcass. Interestingly, with *Nicrophorus* a number of core microbiota do manage to persist despite the ecological instability inherent in this transmission process. This highlights the potential of niche adaptability for these core species, and further suggest a mechanism of bacterial selection based on the antimicrobial properties of the anal secretions, which likely provides a competitive advantage for these microbes to transition onto the carcass. Such a process would ultimately enhance the transmission probability of core microbiome members from adult beetles to their larval offspring, and similar modes of vertical transition are known for other insects, such as Lepidoptera [52]. The results of our study suggest that while transmission of endogenous microbiota by *N*. *defodiens* via anal secretions may not be a universally controlled process, for the bacterial communities that ultimately populate the prepared carcass there is selection both for core microbiota and against some exogenous microbes. These finding are consistent with concepts proposed by other authors [18], and suggest anal secretions used in carcass preservation are integral to a vertical transmission scheme for a niche-adapted core of bacterial species.

## Conclusions

In this study, we sought to evaluate the potential for anal secretions of *N*. *defodiens* to structure the prepared carcass community via transmittance of bacterial taxa from adult beetle digestive tracts to carcasses, where they could also serve as inocula to later influence the gut communities of larval offspring. We found that preparation had a significant effect on the diversity and structure of the carcass bacterial communities when compared to those of the unprepared carcass. While communities of adult digestive tracts, the anal secretions, and prepared carcasses all supported bacterial populations that differed significantly in their structure, we did find evidence supporting the process of microbial ‘seeding’ and ‘weeding’ for *N*. *defodiens*. While controlled transmission of bacteria from adult digestive tracts to prepared carcasses can be mediated by the anal secretions, this process is not a universal phenomenon and some bacterial taxa likely make it into the secretion or prepared carcass communities via niche selection. Potential core bacterial members of the *N*. *defodiens* microbiome, including *Acinetobacter*, *Myroides*, and *Wohlfahrtiimonas*, were recovered in our study, and as previous studies suggest, they likely play essential functional roles in preserving the carcass, assisting with offspring nutrient acquisition, and establishing the digestive tract microbiome in the developing larvae. Further research will be required before functional roles of these core bacterial taxa can be ascertained or successful ‘seeding’ and/or ‘weeding’ of bacterial taxa from the adult to larval gut in *N*. *defodiens* can be more fully evaluated.

## Supporting information

S1 Fig Alphararefaction of observed OTUs. Observed OTUs found in adult digestive tracts (n = 20), the anal secretions (n = 10), on unprepared carcasses (n = 10), and on prepared carcasses (n = 10). Rarefaction curves show the total number of unique OTUs per sample type.
(TIF)

S2 Fig Relative abundances of bacterial genera within different sample types. Bar lengths show the average relative percentage of all sequences for bacterial genera found within each sample type. All genera not specifically identified were assigned to the ‘Other’ category including unidentified bacterial genera.
(TIF)

S1 Table Alpha diversity indices. Alpha diversity metrics for Chao1, Evenness, and Faith’s Phylogenetic Diversity (Faith’s PD). Letters correspond to bacterial communities of specific sample types: A. Reproductive adult digestive tracts and prepared carcasses; B. Anal secretions; C. Unprepared Carcasses. Significance was calculated using pairwise Kruskal-Wallis tests in QIIME v.2.4.
(DOCX)

S2 Table Pairwise PERMANOVA comparisons of bacterial communities between sample types. Bacterial communities in samples were compared in R.3.4.3 using pairwise PERMANOVA analysis with Bonferroni corrections. Pseudo-F values (PF) and P-values (*p*) are provided. Sample types were given acronyms for simplicity: DT–Adult digestive tract, AS–Anal secretions, UC–Unprepared carcass, PC–Prepared carcass.
(DOCX)
