# Supplementary figures and images for: Examining transmission of gut bacteria to preserved carcass via anal secretions in Nicrophorus defodiens
Source: PLoS One. 2019 Dec 2;14(12):e0225711. doi: 10.1371/journal.pone.0225711 (PMC6886834; doi:10.1371/journal.pone.0225711)

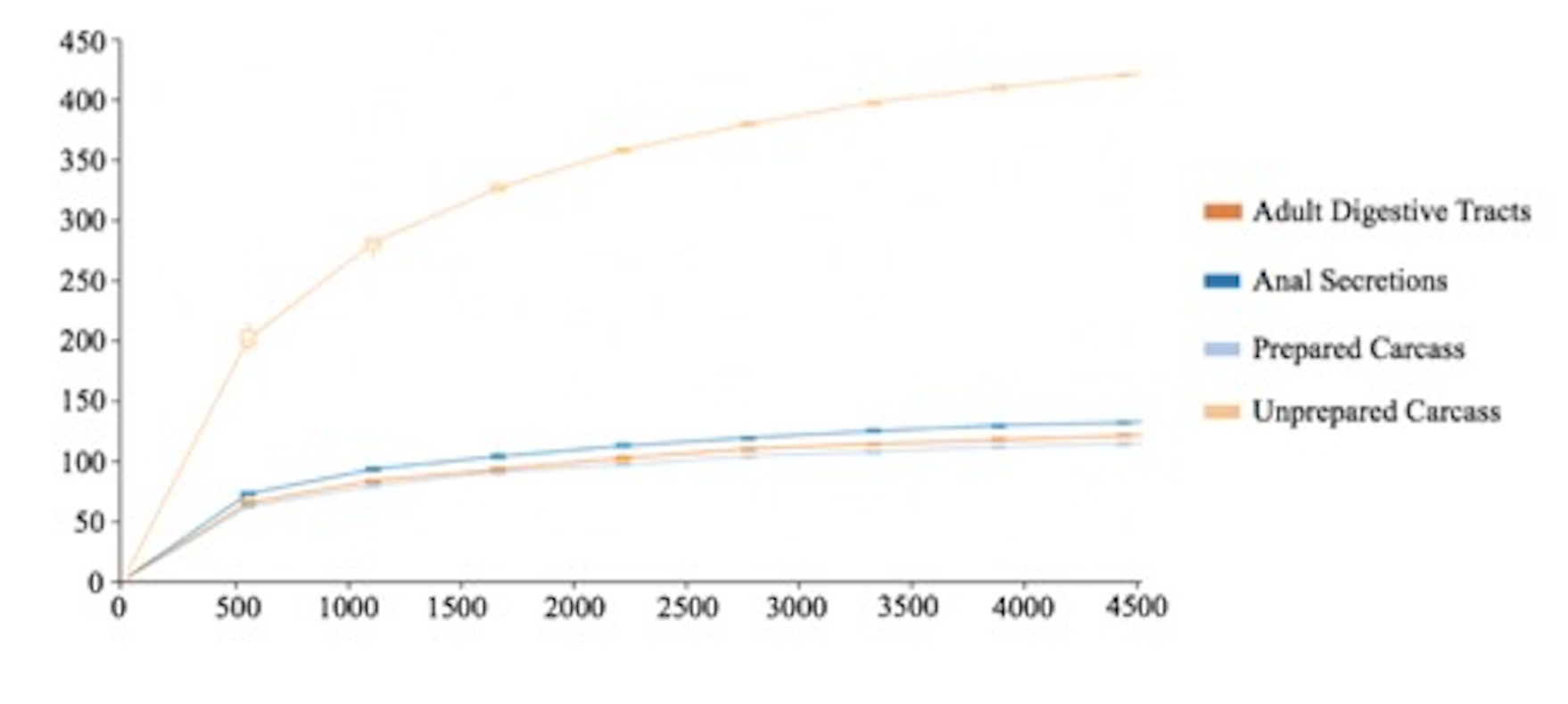

Supplement: S1 Fig — Observed OTUs found in adult digestive tracts (n = 20), the anal secretions (n = 10), on unprepared carcasses (n = 10), and on prepared carcasses (n = 10). Rarefaction curves show the total number of unique OTUs per sample type. (TIF) [file pone.0225711.s001.tif]

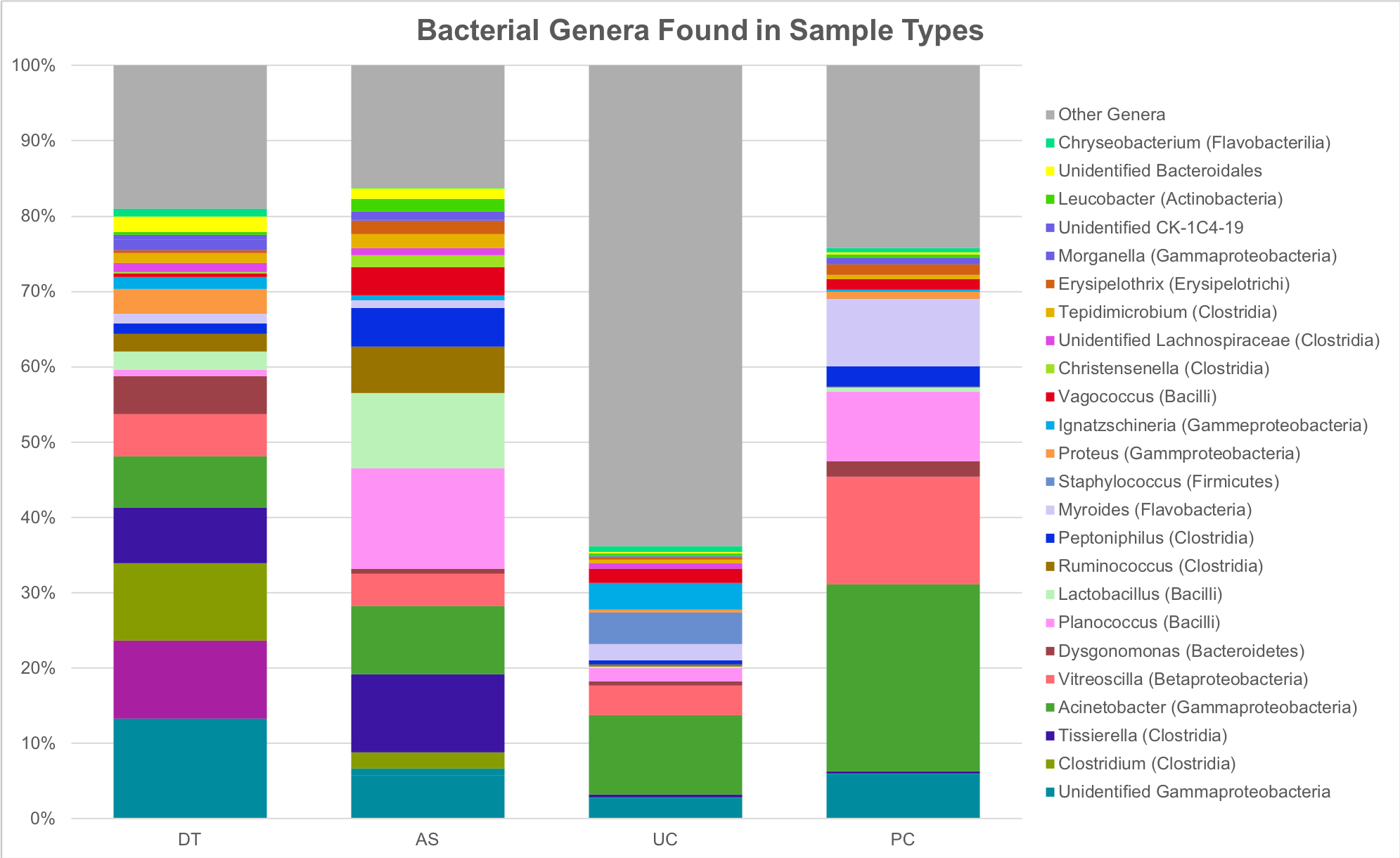

Supplement: S2 Fig — Bar lengths show the average relative percentage of all sequences for bacterial genera found within each sample type. All genera not specifically identified were assigned to the ‘Other’ category including unidentified bacterial genera. (TIF) [file pone.0225711.s002.tif]
