# Supplementary material for: Examining transmission of gut bacteria to preserved carcass via anal secretions in Nicrophorus defodiens
Source: PLoS One. 2019 Dec 2;14(12):e0225711. doi: 10.1371/journal.pone.0225711 (PMC6886834; doi:10.1371/journal.pone.0225711)
Supplement: S1 Table — Alpha diversity metrics for Chao1, Evenness, and Faith’s Phylogenetic Diversity (Faith’s PD). Letters correspond to bacterial communities of specific sample types: A. Reproductive adult digestive tracts and prepared carcasses; B. Anal secretions; C. Unprepared Carcasses. Significance was calculated using pairwise Kruskal-Wallis tests in QIIME v.2.4. (DOCX) [file pone.0225711.s003.docx]

| Alpha Diversity Metric | Comparison | P-value |
| --- | --- | --- |
| Chao1 | A & B | *p* < 0.050 |
| Chao1 | A & C | *p* < 0.001 |
| Chao1 | B & C | *p* = 0.028 |
| Evenness | A & B | *p* < 0.050 |
| Evenness | A & C | *p* < 0.001 |
| Evenness | B & C | *p* = 0.001 |
| Faith’s PD | A & B | *p* < 0.050 |
| Faith’s PD | A & C | *p* < 0.001 |
| Faith’s PD | B & C | *p* = 0.001 |
