# Supplementary material for: Examining transmission of gut bacteria to preserved carcass via anal secretions in Nicrophorus defodiens
Source: PLoS One. 2019 Dec 2;14(12):e0225711. doi: 10.1371/journal.pone.0225711 (PMC6886834; doi:10.1371/journal.pone.0225711)
Supplement: S2 Table — Bacterial communities in samples were compared in R.3.4.3 using pairwise PERMANOVA analysis with Bonferroni corrections. Pseudo-F values (PF) and P-values (p) are provided. Sample types were given acronyms for simplicity: DT–Adult digestive tract, AS–Anal secretions, UC–Unprepared carcass, PC–Prepared carcass. (DOCX) [file pone.0225711.s004.docx]

|  | **DT** | **AS** | **UC** |
| --- | --- | --- | --- |
| **AS** | PF = 55.318  *p* = .001 | --- | --- |
| **UC** | PF = 30.554  *p* = .001 | PF = 14.993  *p* = .001 | --- |
| **PC** | PF = 5.350  *p* = .002 | PF = 14.467  *p* = .001 | PF = 12.699  *p* = .001 |
